# Macroscopic Hematuria Revealing Bladder Myeloid Sarcoma in Acute Myeloid Leukemia

**DOI:** 10.7759/cureus.75912

**Published:** 2024-12-17

**Authors:** Omar Halloumi, Wittnebel Sebastian, Mirvate Harb, Anne Laure Trépant, Fabio Andreozzi

**Affiliations:** 1 Department of Clinical Hematology and Cellular Therapy, Centre Hospitalier Universitaire (CHU) Souss-Massa, Agadir, MAR; 2 Faculty of Medicine and Pharmacy, Université Ibn Zohr, Agadir, MAR; 3 Department of Hematology, Hôpital Universitaire de Bruxelles (HUB) Institut Jules Bordet, Brussels, BEL; 4 Laboratory of Hematology, Le Laboratoire Hospitalier Universitaire de Bruxelles-Universitair Laboratorium Brussel (LHUB-ULB), Brussels, BEL; 5 Department of Pathology, Centre Hospitalier Universitaire (CHU) Erasme, Brussels, BEL

**Keywords:** acute myeloid leukemia, allogeneic stem cell transplantation, extramedullary manifestations, hematuria, myeloid sarcoma

## Abstract

Acute myeloid leukemia (AML) can be presented with extramedullary manifestations, more frequently involving skin and rarely other sites, such as the urinary tract. We report the case of a 37-year-old male patient with a history of testicular cancer who presented to the emergency department with cytopenias and hematuria. Bone marrow analysis diagnosed AML (French-American-British(FAB) classificationM4 subtype, karyotype showing inv16). A diagnostic workup for hematuria revealed a bladder mass that underwent biopsy. Histopathology revealed myeloid sarcoma. The patient was treated with standard induction chemotherapy, followed by consolidation therapy including allogeneic stem cell transplantation, which resulted in complete remission of AML.

## Introduction

Extramedullary manifestations of acute myeloid leukemia (AML) occur in approximately 2.5-9.1% of cases at diagnosis and can also present during relapse [1] and are associated with chromosomal abnormalities such as t(8;21) and inv(16), as well as specific cell surface markers in flow cytometry (CD56, CD2, CD4, CD7) and certain AML subtypes (M2, M4, M5) [1,2]. The most frequently affected sites are the skin and central nervous system (CNS) [3].

We report here the case of a 37-year-old patient with a history of testicular neoplasm, who was diagnosed with AML associated with a myeloid sarcoma of the bladder, clinically revealed by macroscopic hematuria. Urinary bladder involvement is particularly rare and increases the risk of misdiagnosis, as myeloid sarcoma may be confused with a primary urothelial tumor and treated inappropriately [4]. This highlights the importance of a thorough diagnostic workup to guide treatment and improve prognosis.

## Case presentation

A 37-year-old male with a history of non-seminomatous testicular cancer was treated with three cycles of BEP (bleomycin, etoposide, and platinum) chemotherapy (bleomycin 30 mg on days 1, 8, and 15, cisplatin 20 mg/m²/day from days 1 to 5, and etoposide 100 mg/m²/day from days 1 to 5, with one cycle every three weeks), followed by lymph node dissection. He was declared in complete remission, with regular clinical and paraclinical follow-up by his oncologist. Four years later, he presented with profound fatigue, anorexia, and unquantified weight loss, with a rapid progression over one week, without fever or night sweats. He subsequently developed grade II macroscopic hematuria and presented to the emergency department.

Blood tests revealed anemia (hemoglobin 10.1 g/dL), profound thrombocytopenia (platelets 26,000/mcL), neutropenia (neutrophils 540/mcL), and hyperleukocytosis (white blood cells 54,480/mcL), with 24% circulating blasts. Bone marrow evaluation confirmed the diagnosis of acute myeloid leukemia (AML), with myeloid blasts positive for CD33, CD34, CD13, and MPO on flow cytometry, and a significant monocytic component (M4 according to French-American-British (FAB) classification) (Table 1).

**Table 1:** Hematological parameters and FAB classification The reference ranges provided in this table are based on standard clinical reference values commonly accepted for hematological parameters, as reported by the World Health Organization (WHO) and other global health guidelines (2017). FAB: French-American-British

Parameter	Result	Reference Range (WHO)
Hemoglobin (Hb)	10.1 g/dL	13.0–17.0 g/dL (men) / 12.0–15.0 g/dL (women)
Platelets	26,000/mcL	150,000–450,000/mcL
Neutrophils	540/mcL	2,000–7,000/mcL
White Blood Cells (WBC)	54,480/mcL	4,000–10,000/mcL
Circulating Blasts	24%	0% (blasts are normally absent in blood)
Coagulation Tests	Normal	Normal
Myeloid Blasts (CD33+, CD34+, CD13+, MPO+)	Present	Not normally present in peripheral blood
FAB Classification	M4 (Myelomonocytic)	M4: Myelomonocytic acute leukemia (characterized by myeloid and monocytic cell populations)

**Figure 1:**
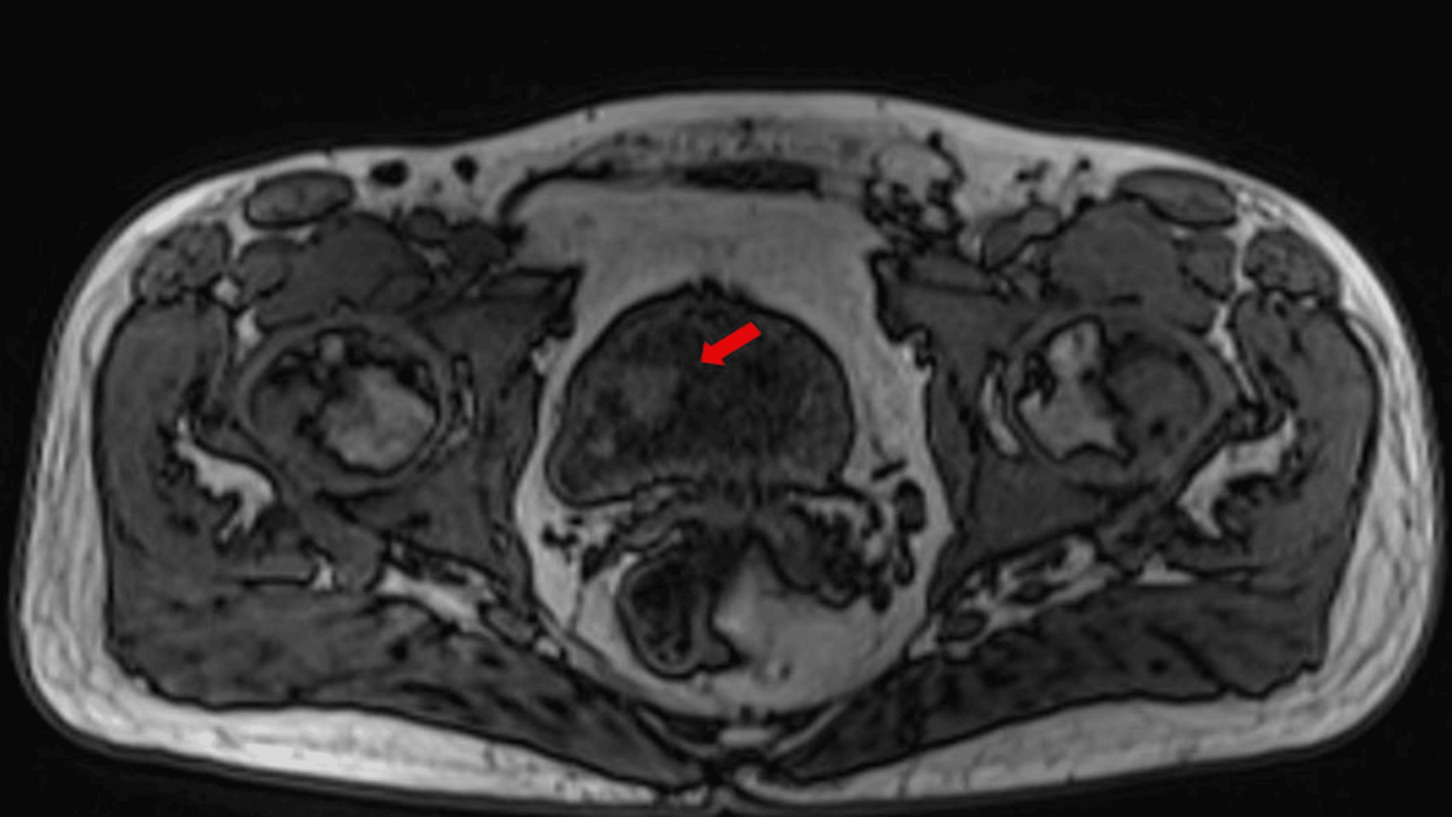
Pelvic MRI showing nodular thickenings of the right bladder wall. Plane: Axial (transverse), passing through the pelvic region; Sequence: Fast Spin Echo

Cytogenetic and molecular analysis revealed inversion 16, a WT1 gene mutation, FLT3 negative. Due to a prior history of testicular cancer and the presence of persisting hematuria, an extensive oncologic work-up was performed. PET-CT didn’t reveal hypermetabolic lesions, while a pelvic MRI showed a nodular bladder wall thickening (Figure 1).

**Figure 2:**
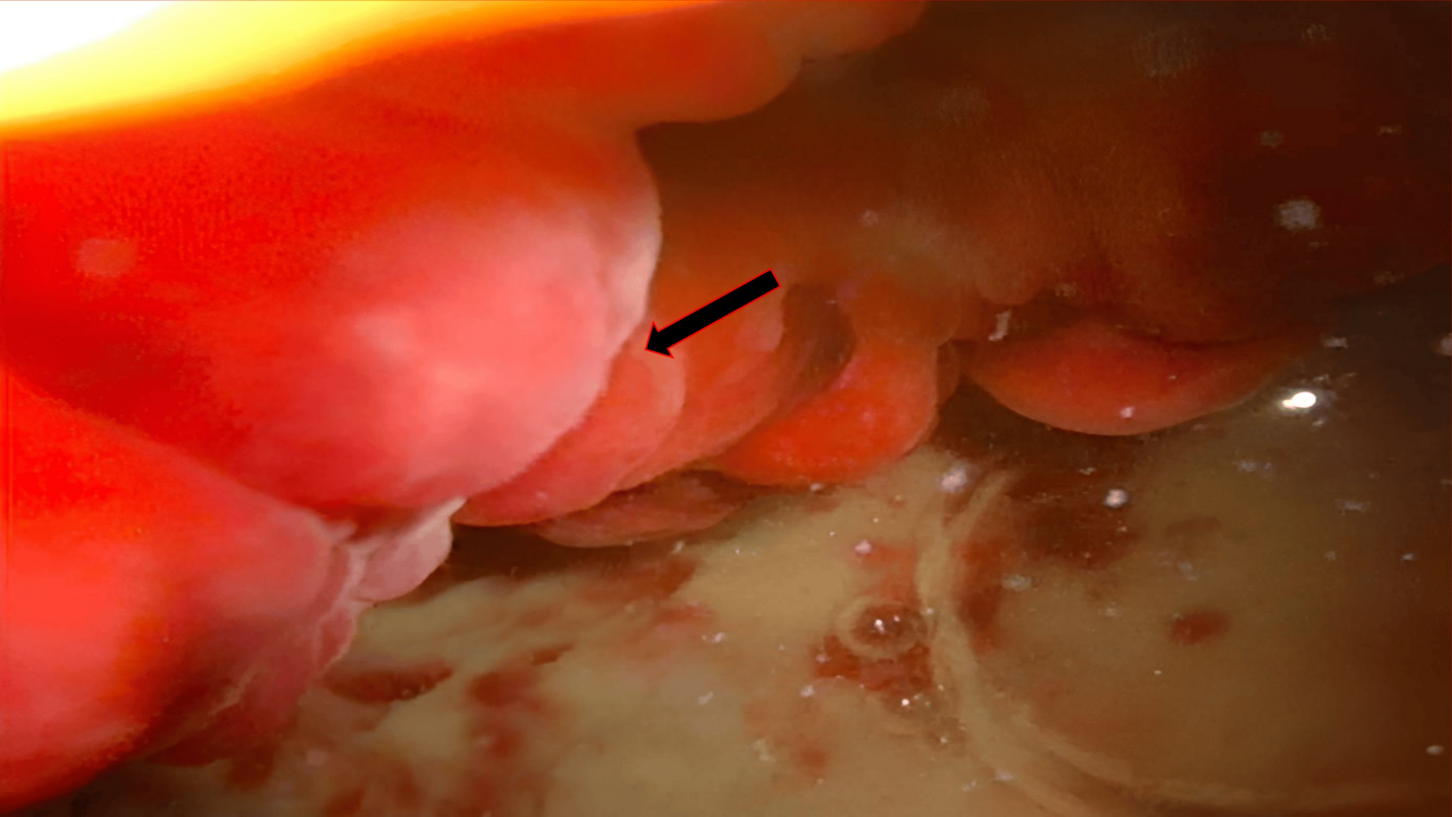
Cystoscopy reveaking a thickened polypoid lesion measuring 3x2x1 cm, located at the dome and right lateral aspect of the bladder, exhibiting friability with contact bleeding.

Urine cytology did not show any neoplastic cells. At cystoscopy, bladder thickening corresponded to a polypoid lesion, measuring 3x2x1 cm, located on the dome and right lateral wall of the bladder, with easy bleeding at contact (Figure 2).

**Figure 3:**
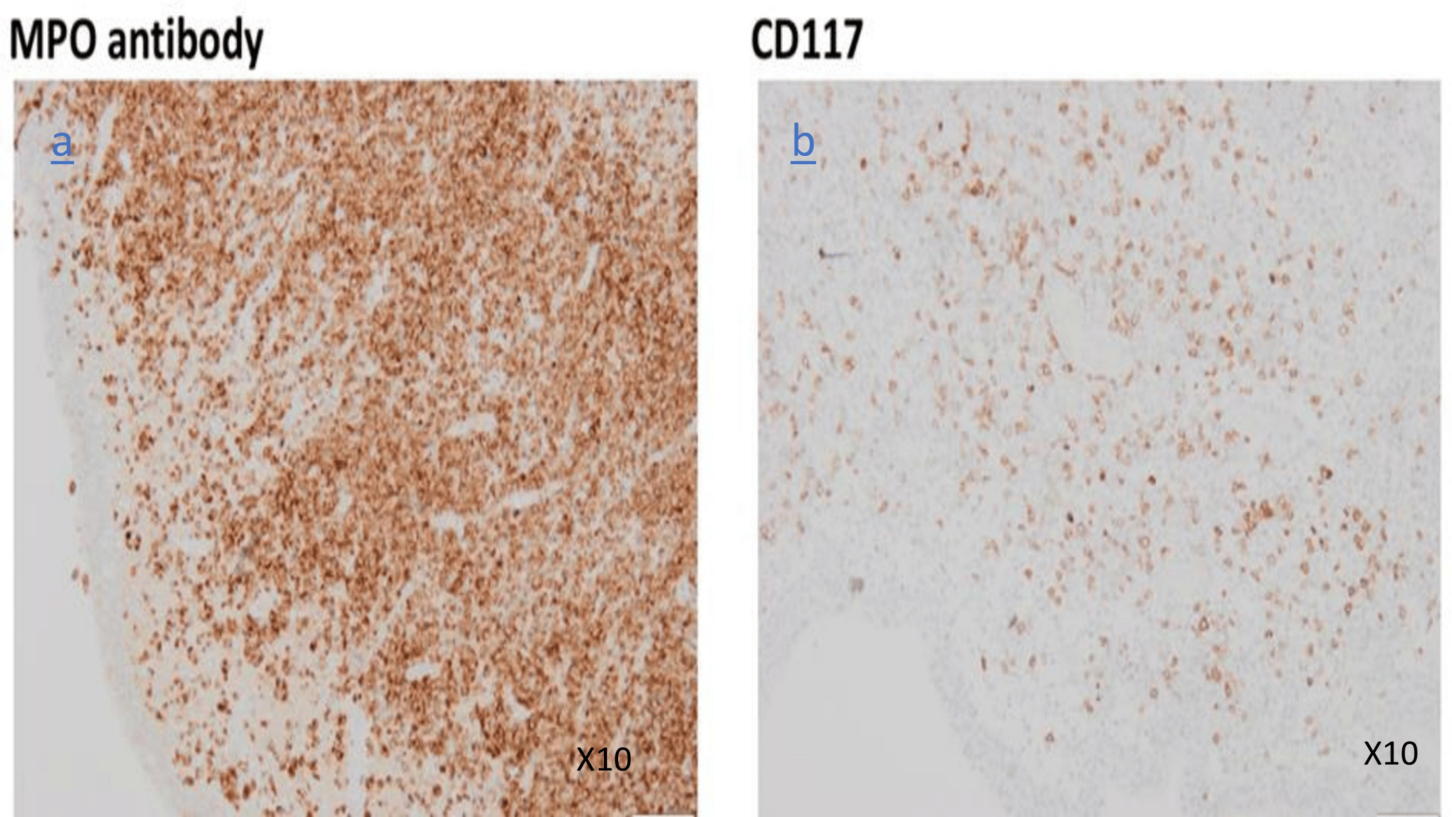
Immunohistochemistry shows (a) diffuse positivity for myeloperoxidase (MPO) and (b) partial positivity for CD117 in blast cells, confirming diffuse infiltration of the bladder wall by myeloid sarcoma. MPO: myeloperoxidase; CD: cluster of differentiation

Histological analysis showed a proliferation of tumor cells arranged in sheets, featuring scant cytoplasm and large, irregular nuclei. Immunohistochemistry showed diffuse positivity for MPO and partial positivity for CD117, confirming diffuse infiltration of the bladder wall by myeloid sarcoma (Figure 3).

The patient underwent standard intensive induction chemotherapy with the 3+7 regimen (three days of daunorubicin 60 mg/m² and seven days of cytarabine 200 mg/m²). Hematuria resolved rapidly after chemotherapy initiation, with improvement in his general condition. On day +25 after induction, the patient was in complete remission, with negative cytological, cytometric, and molecular results, which demonstrated minimal residual disease (MRD) negativity. The patient subsequently received consolidation therapy with intermediate-dose cytarabine, followed by non-familial allogeneic stem cell transplantation (Allo-SCT) with myeloablative conditioning, including total body irradiation and high-dose cyclophosphamide. No further episodes of hematuria were observed during the treatment. Currently, six months after transplantation, the patient remains in complete remission and negative MRD, with regular follow-up.

## Discussion

Extramedullary disease (EMD) in AML is rare and associated with a poor prognosis, particularly in cases of cutaneous involvement [5]. Data on EMD involving non-cutaneous sites, such as the bladder, are limited. Specific cytogenetic abnormalities, such as t(8;21) and inv(16), are frequently associated with EMD, particularly in abdominal localizations [6]. However, bladder involvement remains exceptionally rare, with only 18 documented cases in the literature. These cases highlight the diagnostic challenges and complex therapeutic implications, as bladder involvement can be mistaken for primary urothelial pathology (Table 2) .

**Table 2:** Case reports of myeloid sarcoma involving the urinary tract ADE: Ara-C (cytarabine), daunorubicin, etoposide; AML: acute myeloid leukemia; CRi: complete remission with incomplete hematologic recovery; FISH: fluorescence in situ hybridization; HSCT: hematopoietic stem cell transplantation; MACE: mercaptopurine, Ara-C (cytarabine), cyclophosphamide, etoposide; MDS: myelodysplastic syndrome; MidAC: mitoxantrone, Ara-C (cytarabine); MS: myeloid sarcoma; MVAC: methotrexate, vinblastine, doxorubicin, and cisplatin; NGS: next-generation sequencing; NR: not reported; RAEB: refractory anemia with excess blasts; TCC: transitional cell carcinoma

Case/Year	Age/Sex	Location	Diagnosis	Cytogenetics	Treatment	Status	Time From Diagnosis to Last Follow-Up
Liu et al., 1973 [7]	NR	Bladder	AML	NR	NR	NR	NR
Chaitin et al., 1984 [8]	29 years/F	Bladder trigone	MS	NR	Doxorubicin, vincristine, cytarabine, prednisone	Complete remission	13 months
Cartwright et al., 1991 [9]	16 years/M	Left urethra	AML with MS	NR	External radiation	Death	2 months
Bekassy et al., 1996 [10]	17 years/M	Bladder	AML	NR	Surgery, chemotherapy	Alive	75 months
Aki et al., 2002 [4]	36 years/M	Bladder	Misdiagnosed as TCC then MS	NR	MVAC followed by cytarabine plus doxorubicin	Death	16 days into treatment
Kerr et al., 2002 [11]	80 years/M	Bladder	RAEB relapsing as MS	NR	Local radiation	Recurrence	NR
Hasegeli Uner et al/, 2004 [12]	57 years/F	Urinary bladder cell	MS	NR	Ara-C plus idarubicin and radiation	Complete remission	1 month
Al-Quran et al., 2006 [13]	47 years/M	Bladder trigone and right epididymis	Poorly differentiated, neoplasm, MS	Bone marrow 47XY, inv(16), +22; bladder inv(16) by FISH	Ara-C plus idarubicin	Complete remission	32 months
Kong et al., 2010 [14]	NR	Urinary bladder	MS	NR	NR	NR	NR
Geok Chin et al., 2011 [15]	70 years/F	Urinary bladder and abdominal wall	AML with MS	Bone marrow AML	NR	NR	NR
John et al., 2013 [16]	39 years/F	Bladder mesentery	MS	Bone marrow 46XX, inv(16)	Ara-C plus idarubicin, Ara-C, and consolidation	Complete remission	8 months
Delhi Kumar et al., 2014 [17]	18 months/F	Bladder, Bilateral Retro-orbital	AML with MS	NR	Induction Chemotherapy	Death	1 month
Grantham et al., 2015 [18]	56 yeats/M	Bladder	MS with RAEB-2	NR	NR	NR	NR
Pearson et al., 2021 [19]	70 years/M	Bladder	Secondary AML to MDS and MS	Normal male karyotype, with NGS: FLT3-ITD, IDH2, and RUNX1 mutations.	Venetoclax and cytarabine, followed by multiple cycles of infusional cytarabine.	Death	24 months
Mandhan et al., 2021 [20]	63 years/M	Bladder	MS	Complex cytogenetics (monosomies 18, 19, 21; 5q/7q abnormalities), TP53 mutation (34%)	Decitabine, Venetoclax, allogeneic HSCT	Complete remission	Day +60 post-transplantation
Tuna et al., 2022 [21]	12 years/F	Right side of the bladder	AML and MS	Bone marrow t(8; 21)	(ADE, MACE, MidAC), transurethral resection, radiotherapy, allogeneic HSCT	Complete remission	More than 5 years
Lucijanic et al., 2022 [22]	75 years/M	Bladder	MDS with excess blasts-2, and MS, Secondary AML	Normal	Initial Azacitidine, followed by Azacitidine+Venetoclax	Achieved CRi, later progression to AML and Death	37 months
Huang et al., 2024 [23]	48 years/M	Bladder	MS	NR	MidAC	Complete remission	Currently under follow-up; autologous transplantation planned
Current case, 2024	37 years/M	Bladder (dome and right lateral wall)	AML and MS	Inv( 16), WT1 mutation,	daunorubicin and cytarabine), consolidation with intermediate-dose cytarabine, allogeneic HSCT	Complete remission	8 months

In the current case, the patient presented with gross hematuria, associated with a history of non-seminomatous testicular cancer treated with the BEP regimen, which led to an extensive diagnostic workup revealing bladder involvement by myeloid sarcoma. The BEP protocol, which includes etoposide, bleomycin, and cisplatin, is known to be associated with long-term complications, particularly the development of secondary acute leukemia. This complication occurs with an incidence ranging from 2% to 12%, typically within two to three years after treatment, implying the need for long-term clinical and biological monitoring in these patients [24].

Despite AML with inv(16) being typically associated with a favorable prognosis in de novo cases, the combination of secondary AML and complex extramedullary involvement, including the rare bladder localization, led us to opt for an aggressive treatment approach [25].

We chose consolidation treatment with Allo-SCT, as a potential curative strategy. Total body irradiation (TBI) was included as part of the conditioning regimen due to its capacity to effectively target extramedullary sites, including potential sanctuary areas that are less accessible to systemic chemotherapy.

This approach, combined with rigorous follow-up, resulted in complete remission with negative MRD, highlighting the importance of personalized management for such a rare and complex clinical presentation.

## Conclusions

This case highlights the importance of a thorough evaluation of hematuria, especially in patients with a history of cancer, emphasizing the need for long-term clinical and biological monitoring to detect potential complications and ensure optimal management. It also underscores the necessity of considering myeloid sarcoma of the urinary tract as a diagnostic possibility. Due to its rarity, this condition can easily be overlooked, but early detection is crucial for tailoring therapeutic strategies and improving clinical outcomes in such rare and complex presentations.
